# Ethnic inequalities and trends in stunting prevalence among Guatemalan children: an analysis using national health surveys 1995–2014

**DOI:** 10.1186/s12939-019-1016-0

**Published:** 2019-07-18

**Authors:** Giovanna Gatica-Domínguez, Cesar Victora, Aluisio J. D. Barros

**Affiliations:** 1 International Center for Equity in Health, Federal University of Pelotas, Marechal Deodoro 1160, 3rd floor, Pelotas, RS Brazil; 2 Federal University of Pelotas, Pelotas, Brazil

**Keywords:** Stunting, Health equity, Health status disparities, Ethnic groups, Guatemala

## Abstract

**Background:**

Guatemala has the highest prevalence of stunting among under-five children in Latin America. We aimed to compare indigenous and non-indigenous under-five child populations in relation to stunting, as well as to explore the intersectionality of ethnicity by wealth and by place of residence. We also studied how the ethnic inequalities changed over time, using five ENSMI surveys from 1995 to 2014.

**Methods:**

Five national health surveys carried out between 1995 and 2014 were analysed. World Health Organization (WHO) 2006 growth standards were used to calculate stunting prevalence. Self-reported ethnicity was classified as indigenous or nonindigenous. Wealth was measured through an asset-based index, and households were classified into quintiles (for analyses of the whole populations) or tertiles (for analyses of intersectionality with ethnicity). Area of residence was recorded as urban or rural, according to country definition.

**Results:**

Overall stunting prevalence declined by 9.8 percentage points (95% CI −16.4 to − 3.3) from 1995 to 2014. The slope index for absolute inequalities in stunting - which corresponds to the difference in prevalence between the wealthiest and poorest households - ranged from − 52.9 to − 60.4 percentage points, with no significant change over time. Children in rural areas were consistently more stunted than those in urban areas, but rural indigenous children were significantly worse than any other group. Indigenous children in the poorest tertile of family wealth consistently presented the highest stunting prevalence, compared to all other groups. Time trends in stunting were assessed through the average annual absolute change (AAAC). The fastest decline was observed among indigenous children from the middle wealth tertile (AAAC = − 1.21 percentage points per year (pp/y); 95% CI − 1.45 to − 0.96) followed by nonindigenous children also from the middle tertile (AAAC = − 0.80 pp./y; 95% CI − 0.99 to − 0.60). Stunting prevalence in the two poorest tertiles of indigenous children in 2015 was similar to what nonindigenous children presented in 1995, 20 years earlier. In the wealthiest tertile, indigenous children were far worse off than nonindigenous children 20 years earlier.

**Conclusions:**

In terms of stunting prevalence, poor and rural indigenous children are twenty years behind nonindigenous children with similar characteristics.

**Electronic supplementary material:**

The online version of this article (10.1186/s12939-019-1016-0) contains supplementary material, which is available to authorized users.

## Background

Childhood stunting is the impairment in linear growth that mostly occurs during the first 1,000 days of life, a critical period from conception to the second birthday. Its primary causes include poor maternal health and nutrition, persistent exposure to poor nutrition due to inadequate infant and young child feeding practices, and recurrent infections [1–5], with poverty acting as a distal determinant [3, 6]. Childhood stunting is considered by many to be the best overall indicator of child welfare, representing an accurate marker of poor child development and social inequalities [4, 7]. The long-term consequences of stunting include shorter adult height, lower intelligence and school achievement, and reduced economic productivity that contribute to perpetuating the vicious cycle of undernutrition, poverty, and inequality [3, 6].

Guatemala is a multicultural and multilingual country with a population of nearly 17 million people [8] that presents the sixth highest stunting prevalence in the world, and the highest in Latin America [6]. After Bolivia, it is the country with the highest proportion of indigenous population in the region; with 46% of the population belonging to one of 23 officially recognized ethnolinguistic groups [9]. In 2014, Guatemalan gross domestic product (GDP) per capita was 7,147 international dollars (constant 2011, purchasing power parity). Poverty levels are high, with 59.3% of the population under the national poverty line and 8.7% under the extreme poverty line [8, 10]. In addition, Guatemala is one of the countries from Latin America with the highest levels of income inequality, where indigenous and rural populations live under multidimensional poverty, suffering from inadequate access to basic sanitation services, low education, as well as lack of productive assets and formal employment [11]. Indigenous people still live in social exclusion partly due to the language in which public services are provided and partly because the national budget allocation does not take their needs into account, resulting in smaller proportional allocations to areas with large indigenous populations [12].

For 30 years, the Ministry of Public Health and Social Assistance has carried out the National Maternal and Child Health Surveys (ENSMI, acronym in Spanish) with technical and financial support from international cooperation agencies, governmental and non-governmental institutions. These surveys were designed to address the poor quality and coverage of national health information systems and have become an official data source for maternal and child health. The surveys also allow benchmarking of maternal and child health indicators, because they have the quality standards of international survey initiatives – Demographic Health Surveys (DHS) and Reproductive Health Surveys (RHS).

In 2014, Ramírez-Zea et al. analyzed 1998, 2002 and 2008 ENSMI surveys and showed that indigenous children were more stunted than non-indigenous children in the three time points, although in both ethnic groups there was a reduction in the stunting prevalence [13]. In 2014, ENSMI reported that the national-level stunting prevalence in children under-five was 46.5% [9]. Other reports have shown that, as in most other countries, the stunting in Guatemala is more prevalent among poor children and those living in rural areas [14, 15].

The importance of stunting has been recognized when the World Health Organization (WHO) adopted the resolution of the World Health Assembly (WHA) in 2012, which endorsed six global nutrition targets, and the first one states *“a reduction of 40% in the number of under-five children who are stunted by 2025, compared to the baseline of 2010”* [16]. Following the same rationale, it was estimated a relative reduction of 50% [8] was necessary in order to achieve target 2.2.1 of the Sustainable Development Goals (SDG) by 2030 about stunting prevalence [17]. In addition, SDG 17.18 stresses the need for national statistics stratified according to ethnicity (https://sustainabledevelopment.un.org/), since it is one among other factors in which social inequalities are manifested [18]. Socioeconomic factors interacts in complex ways with ethnicity that influence on child stunting [19]. Household wealth is considered the most important determinant which explains the level and inequalities in stunting, but also place of residence is considered important because it is a proxy of access to public services, education and wealth [20, 21]. These global targets need to be translated into context-specific national targets, taking into consideration present stunting levels and recent trends [22]. We aimed to compare indigenous and non-indigenous under-five child populations in relation to stunting, as well as to explore the intersectionality of ethnicity by wealth and by place of residence. We also studied how the ethnic inequalities changed over time, using five ENSMI surveys from 1995 to 2014.

## Methods

The 1995, 1998 and 2014 ENSMI surveys were part of the Demographic Health Surveys or DHS initiative (https://dhsprogram.com), whereas 2002 and 2008 ENSMI surveys of the Reproductive Health Surveys or RHS initiative (https://www.cdc.gov/reproductivehealth/global/tools/surveys.htm). Although these are different global survey programs, both were designed to produce highly comparable data, including questionnaires and measurement protocols. All the ENSMI are household surveys, nationally representative and based on cluster samples, with a focus on reproductive, maternal, newborn and child health (RMNCH). Detailed descriptions of sampling and data collection methods for each of the ENSMI surveys are presented elsewhere [9, 23–27].

The 1995, 1998 and 2014 ENSMI datasets were obtained from DHS website (https://dhsprogram.com/data/available-datasets.cfm), and 2002 and 2008 ENSMI dataset were downloaded from The Institute for Health Metrics and Evaluation through the Global Health Data Exchange website (http://ghdx.healthdata.org/series/reproductive-health-survey-rhs). All estimates are based on our own analyses of the raw datasets in order to obtain standardized estimates allowing comparability across surveys.

### Stunting

In addition to intervention coverage indicators, all surveys obtained anthropometric measures on children under-5 years of age. Length (for children under 24 months of age) and height (for children between 24 and 59 months) was obtained. Locally-made measuring boards were used in 1995 and “ShorrBoards®” (Weigh and Measure, LLC, Olney, Maryland, USA) in the later surveys by trained examiners, who were standardized using the Habicht method [9, 24–27]. Accordingly with the international agreement, we classified under-five children as stunted if they fell below − 2 standard deviations (SD) from the median of the length/height-for-age Z score, using the WHO 2006 Child Growth Standards [7, 28].

Prior to calculating stunting prevalence, extreme values in the length/height-for-age Z score were flagged because probably those values are due to measurement or data-entry errors, and they were eliminated in the calculation. The cutoff values used to flag the data and clean the datasets (a score Z < − 6 or > 6) were specified as part of the WHO (2006) growth standards [28]. As an internal quality control, we compared our estimates of national-level stunting prevalence with the published ​results in the survey official report.

### Ethnicity

Self-reported ethnicity was obtained from the child’s mother or caretaker, using following question: “Usted ¿cómo se considera: maya, ladino/mestizo, garífuna, xinca o de otra etnia?” (how do you consider yourself? Mayan, mixed race, garífuna, xinca or another ethnicity). Ladinos are defined as those with mixed European and indigenous ethnicity, and garifunas are afrodescendants. In the present analyses, ethnicity was dichotomized as indigenous (Mayan or xinca) or nonindigenous (all others).

### Inequality stratifiers

Urban and rural residence was based on the classification of the sampled clusters by the national government at the time of the survey. We used the household asset score provided with DHS-type ENSMI surveys as an indicator of wealth, classifying the households into quintiles. The asset score uses information on household assets, building materials and utilities like water and electricity. It is generated by principal component analysis and adjusted for urban and rural residence according to a methodology developed by DHS [29]. For RHS-type ENSMI surveys we calculated the wealth score ourselves using the exact same DHS methodology. Because the classification into quintiles is done at household level, and fertility is higher among the poor, the number of children in the poorest quintile tends to be more than 20% of all children surveyed. Conversely, the wealthiest quintile generally has less than one-fifth of all children. Given limitations in sample size when looking at ethnicity subgroups, we used an alternative classification of households in tertiles for some analyses. Urban or rural area of residence was classified according to the local authorities.

### Data analysis

We described stunting prevalence according to ethnicity, household wealth, and place of residence. To summarize wealth-related inequality, we used the slope index of inequality (SII) which provides the difference in percentage points (pp) between the fitted values ​of the top and the bottom of the wealth distribution. The SII was calculated through a logistic regression model which uses the natural logarithm of the odds of stunting prevalence as the outcome and the wealth index as the independent variable [30]. We also present a double stratification of stunting according to ethnicity and inequality stratifiers. Absolute differences for each category resulting from the combination of ethnicity and area of residence, and ethnicity and wealth tertiles, were calculated by subtracting stunting prevalence in 2014 from that in 1995. We considered a *p* value < 0.05 as an interaction statistically significant. Variance-weighted least squares regression was used to estimate the average annual reduction of stunting by ethnic group and household wealth tertiles. All analyses considered the survey design, including sampling weights, clustering and stratification were performed in Stata 15 (StataCorp, College Station, TX, USA) [31].

### Ethics

We used publicly available data from national surveys in our analyses, and ethical issues were dealt with by the institutions carrying out the surveys. Further information about the surveys and ethics can be found in the published national reports [9, 23–27].

## Results

### Ethnicity distribution by place of residence and wealth tertiles

**Fig. 1:**
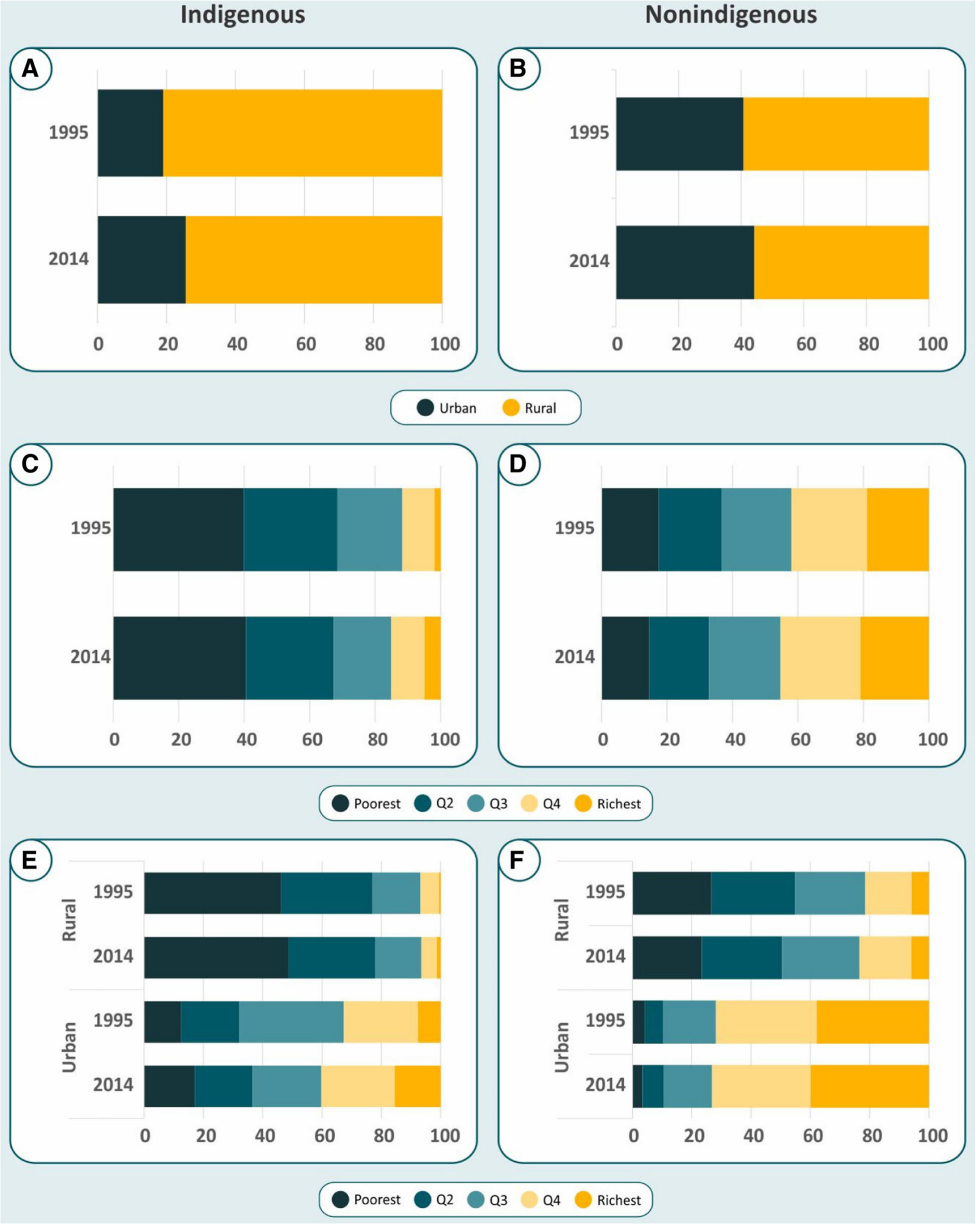
Distribution of under-five indigenous and nonindigenous children according to area of residence (A and D), wealth quintiles (B and E), area of residence and wealth quintiles (C and F)

Figure 1a–e and Additional file 1: Table S1, Table S2, and Table S3 show the distribution of indigenous and nonindigenous children according to urban-rural residence, wealth quintiles and the combination of wealth and residence. The proportion of indigenous children living in rural areas was 80.9% in 1995, decreasing slightly to 74.4% in 2014 (Fig. 1a), whereas the proportions of rural nonindigenous children were lower: 59.2% in 1995 and 55.8% in 2014 (Fig. 1b). Indigenous children were always poorer than nonindigenous children (Fig. 1c–d) although there were slight increases in the proportion of indigenous children in the wealthiest quintiles over time whereas the proportion in the poorest quintiles remained unchanged. Also, no relevant changes in the distribution of wealth were observed among nonindigenous children (Fig. 1d). Looking at both area and wealth, poverty is much more common in rural than in urban areas. The patterns of urban and rural distribution of children according to wealth did not change over time (Fig. 1e–f), except for a possible increase in the proportion of urban indigenous children belonging to the poorest and wealthiest quintiles (Fig. 1e). This finding should be interpreted with caution due to the small urban indigenous population (Fig. 1a).

### Trend of stunting prevalence according to socioeconomic factors

The proportion of the indigenous children varied slightly from one survey to another: 42.7% (1995), 38.2% (1998), 41.6% (2002), 46.2% (2008), and 46.7% (2014). Under-five children sample sizes in the surveys varied from 3,538 in 1998 to 12,567 in 2014, partly due to differences in the sampling methodology (Table 1). Most children lived in rural areas (from 68.5% in 1995 and 64.5% in 2014), and about half were from the poorest tertile (from 45.8% in 1995 and 49% in 2014).

**Table 1:** Stunting prevalence in under-five children according to ethnic group, place of residence and wealth quintiles

Stratifiers	DHS 1995 (*N* = 7809)	DHS 1998 (*N* = 3538)	RHS 2002 (*N* = 4623)	RHS 2008 (*N* = 8483)	DHS 2014 (*N* = 12567)
	% (95% CI)	% (95% CI)	% (95% CI)	% (95% CI)	% (95% CI)
Ethnic group					
Indigenous	72.7 (70.5; 74.7)	73.6 (70.0; 76.9)	75.0 (72.8; 77.0)	63.6 (61.8; 65.5)	61.1 (58.8; 63.5)
Nonindigenous	43.0 (39.9; 46.1)	42.1 (37.5; 47.0)	41.2 (38.7; 43.8)	34.9 (33.2; 36.7)	33.9 (31.9; 35.9)
*Difference (pp)*	29.7	31.5	33.8	28.7	27.2
Place of residence					
Rural	62.6 (60.6; 64.6)	61.8 (56.9; 66.3)	61.1 (58.9; 63.2)	56.7 (55.1; 58.3)	53.0 (50.8; 55.1)
Urban	40.2 (36.0; 44.5)	39.2 (30.6; 48.6)	42.1 (38.7; 45.6)	33.2 (31.1; 35.3)	34.6 (31.9; 37.4)
*Difference (pp)*	22.4	22.6	19.0	23.5	18.4
Wealth quintiles					
Poorest	70.5 (67.7; 73.1)	71.3 (66.0; 76.2)	74.1 (71.7; 76.3)	67.9 (65.8; 69.9)	65.9 (63.3; 68.4)
2nd	67.2 (64.4; 70.0)	69.0 (64.5; 73.2)	64.9 (61.3; 68.3)	57.7 (55.1; 60.3)	57.1 (54.4; 59.8)
3rd	58.9 (55.3; 62.5)	58.2 (49.2; 66.6)	47.4 (43.6; 51.2)	41.7 (38.7; 44.8)	43.8 (41.0; 46.6)
4th	39.3 (34.2; 44.7)	34.0 (28.6; 40.0)	40.0 (35.0; 45.2)	24.8 (21.8; 28.2)	28.9 (26.4; 31.4)
Wealthiest	15.8 (12.2; 20.4)	15.0 (11.1; 20.1)	18.1 (12.8; 24.8)	14.3 (11.2; 18.0)	17.4 (15.1; 19.9)
*SII (pp)*	*−52.9 (−58.4; − 47.4)*	*− 60.4 (− 68.6; − 52.3)*	*− 57.2 (− 63.3; − 51.1)*	*−60.2 (− 64.2; − 56.1)*	*−55.8 (− 59.7; − 51.9)*
Total	55.6 (53.5; 57.6)	54.0 (49.8; 58.1)	54.9 (53.0; 56.7)	48.0 (46.7; 49.4)	46.5 (44.8; 48.2)

*SII* Slope index of inequality (absolute inequality)

Overall stunting prevalence declined 9.8 pp. (95% CI -16.4; − 3.3) from 55.6% in 1995 to 46.5% in 2014, with virtually no change between 1995 and 2002 (Table 1 and Additional file 1: Table S4). In every survey, stunting was more common for indigenous children than for nonindigenous ones: 72.7% vs 43.0% in 1995, down to 61.1% vs 33.9% in 2014. Children in rural areas also presented more than a 50% excess in stunting compared to urban children. Regarding family wealth, there were monotonic inverse associations with stunting prevalence in all five surveys, with children from the poorest quintile presenting four-fold increases relative to those in the wealthiest quintiles. In terms of absolute wealth inequalities, the average slope index was no less than 57 pp, with no evidence of a reduction over time (test for trend in the slope index, *P* = 0.8).

### Trend of stunting by ethnicity and place of residence

Table 2 and Additional file 1: Table S4 show analyses of the intersectionality between ethnic group and area of residence, and between ethnic group and wealth tertiles (instead of quintiles to ensure adequate sample sizes). For both ethnic groups, children in rural areas were consistently more stunted than those in urban areas, but the rural indigenous children were significantly worse than any other group. The rural-urban gaps were much wider for nonindigenous than for indigenous children. Urban indigenous children had the largest reduction in stunting prevalence over time (− 15.3 pp). Although indigenous and nonindigenous children from rural areas had intermediate reductions in the stunting prevalence over time (− 11.1 pp. and − 9.8 pp., respectively). Urban nonindigenous children had the smallest improvement over time (− 5.2 pp).

**Table 2:** Prevalence of stunted indigenous and nonindienous children under-five according to place of residence and wealth tertiles

Ethnic group	Year	Place of residence		Wealth tertiles		
		Rural % (95% CI)	Urban % (95% CI)	Poorest % (95% CI)	Middle % (95% CI)	Wealthiest % (95% CI)
Indigenous	1995	73.6 (71.4; 75.7)	68.7 (62.5; 74.3)	74.3 (71.7; 76.7)	73.4 (70.0; 76.4)	46.4 (35.5; 57.6)
	1998	74.1 (69.9; 77.8)	71.8 (62.7; 79.3)	73.7 (69.3; 77.7)	76.5 (68.4; 83.0)	47.9 (25.4; 71.3)
	2002	78.3 (76.2; 80.2)	65.5 (59.9; 70.5)	80.4 (78.4; 82.3)	68.6 (63.5; 73.2)	45.9 (36.6; 55.5)
	2008	67.7 (65.5; 69.8)	52.3 (48.8; 55.8)	70.7 (68.5; 72.8)	58.3 (54.8; 61.8)	33.9 (28.2; 40.2)
	2014	63.8 (61.0; 66.5)	53.4 (48.8; 58.0)	67.1 (64.3; 69.7)	51.5 (47.7; 55.3)	34.8 (27.7; 42.7)
*Absolute difference (pp)*		*−9.8*	*−15.3*	*−7.2*	*−21.9*	*−11.6*
Nonindigenous	1995	51.6 (48.1; 55.0)	30.6 (25.6; 36.0)	62.9 (59.3; 66.3)	44.7 (41.4; 48.1)	18.9 (15.6; 22.8)
	1998	51.4 (45.2; 57.5)	29.9 (22.4; 38.6)	66.2 (60.6; 71.4)	42.4 (36.9; 48.1)	17.7 (13.4; 22.8)
	2002	47.2 (44.0; 50.4)	31.2 (27.3; 35.3)	59.9 (56.2; 63.4)	37.2 (33.3; 41.3)	20.1 (15.7; 25.2)
	2008	44.2 (41.8; 46.6)	23.9 (21.4; 26.6)	56.5 (53.4; 59.5)	37.1 (34.1; 40.2)	17.7 (15.1; 20.6)
	2014	40.5 (37.7; 43.3)	25.4 (22.9; 28.0)	52.9 (49.6; 56.2)	28.9 (26.7; 31.3)	14.3 (12.1; 16.8)
*Absolute difference (pp)*		*−11.1*	*−5.2*	*−10.0*	*−15.8*	*−4.6*

*Absolute difference* Stunting prevalence in 2014 - stunting prevalence in 1995 for each subgroup

### Trend of stunting by ethnicity and wealth tertiles

Poor indigenous children presented consistently the highest stunting prevalence, compared to other groups (Table 2 and Additional file 1: Table S5). Comparing poor indigenous children with rich nonindigenous children in the same survey, the differences in stunting prevalence were always greater than 50 pp, as around three quarters of the former were stunted, compared to less than 20% of the latter. For both indigenous and nonindigenous children, those belonging to the middle tertile had the largest reduction in stunting prevalence over time compared to other children in the same ethnic group (− 21.9 pp. and − 15.8 pp., respectively) Among indigenous, those from the first tertile had the lowest reduction (− 7.2 pp). Indigenous children from the third tertile and nonindigenous children from the first tertile presented intermediate reductions in stunting prevalence over time (− 11.6 pp. and − 10.0 pp., respectively). Nonindigenous from the wealthiest tertile, where prevalence was relatively low in 1995, had the lowest stunting reduction over time (− 4.6 pp). There was strong statistical evidence of an interaction between ethnicity and rural residence (*p* < 0.001), and between ethnicity and wealth (*p* < 0.001).

### Average annual absolute change in stunting by ethnicity and wealth tertiles

**Fig. 2:**
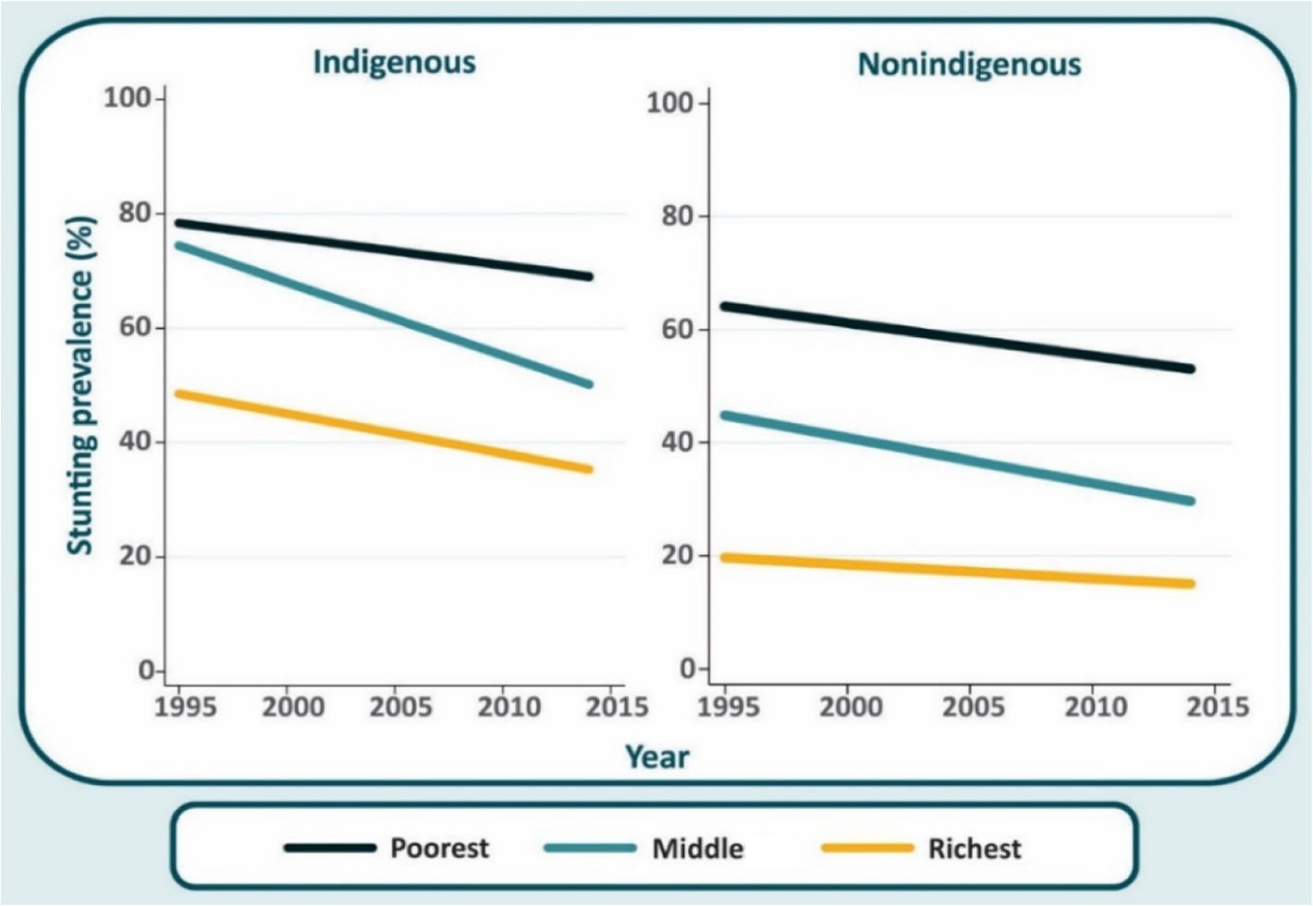
Average annual reduction in stunting prevalence among indigenous and nonindigenous by wealth tertiles

The average annual absolute change (AAAC) in stunting by ethnicity group and wealth tertiles calculated through regression models is presented in Fig. 2. The fastest reduction in stunting was observed among indigenous children from the middle tertile (AAAC = − 1.21 percentage points per year (pp/y); 95% CI − 1.45 to − 0.96) followed by nonindigenous children also from the middle tertile (AAAC = − 0.80 pp./y; 95% CI − 0.99 to − 0.60) and indigenous children from the wealthiest tertile (AAAC = − 0.77 pp./y; 95% CI − 1.41 to − 0.13), respectively. Nonindigenous children from the wealthiest tertile had the lowest average annual reduction in stunting prevalence over time (AAAC = − 0.25 pp./y; 95% CI − 0.45 to − 0.05). Among children from the poorest tertile, indigenous had similar average annual reduction (AAAC = − 0.48 pp./y; 95% CI − 0.66 to − 0.31) than nonindigenous (AAAC = − 0.58 pp./y; 95% CI − 0.81 to − 0.35). Figure 2 also makes very clear that the stunting prevalence in the two poorest tertiles of indigenous children in 2015 is similar to what nonindigenous children presented in 1995, 20 years earlier. Among the wealthiest group, indigenous children are way worse than nonindigenous children 20 years earlier.

## Discussion

In almost 20 years, stunting prevalence decreased ~ 10 pp. in Guatemalan indigenous and nonindigenous children. Given the very high prevalence observed in 1995, the magnitude of reduction is rather small, compared to other countries in the region. Urban indigenous children had the highest stunting prevalence reduction over time (− 20.2 pp. over the period). In addition, indigenous children from the second tertile had the highest average annual reduction in stunting prevalence (1.21 pp. per year) followed by nonindigenous children from the second tertile (0.8 pp. per year). In almost 20 years, despite the reduction observed in stunting, indigenous children in 2014 present a higher stunting prevalence than their nonindigenous counterparts in 1995.

Previous reports, which used data of national surveys, compared the stunting prevalence among several Latin American countries, including Guatemala, and they showed that indigenous children are more likely to be stunted than non-indigenous children [14, 32, 33]. Ramírez, et al. described time trends by ethnicity using national representative surveys of Guatemala from 1998 to 2008 [13]. The authors reported higher prevalence of stunting among indigenous children compared to non-indigenous, with prevalence decreasing over time in both groups. Comparing to our study, they did not investigate intersectionality between ethnicity and socioeconomic factors, and did not have the 1995 and 2014 surveys.

The absolute reduction in stunting prevalence observed in this study was similar with those reported in single-country reports on Bolivia, Peru and Mexico, which have large indigenous populations, but with lower national stunting prevalence than Guatemala. For example, Bolivia with a larger indigenous population than Guatemala, reduced stunting prevalence by 5.7 pp. in 10 years, Mexico 13.3 pp in 24 years and Peru 18.5 pp. in 20 years [33–36]. The substantial reduction in stunting in Peru may be explained by the adoption of anti-poverty policies and sustained implementation of equitable crosscutting interventions, with focus on poorest areas [35, 37].

Then etiology of stunting is complex, involving social, economic, political and cultural factors as distal determinants, according to the widely-adopted UNICEF framework for the social determinants of child undernutrition [6]. Considering that ethnicity is strongly associated with socioeconomic status in Latin America, it may be regarded as one of the distal determinants of stunting - as made evident in the present analyses – and an important contributor to the intergenerational transmission of poor nutrition and poverty [38]. In the case of Guatemala, it is observed that the maps of extreme poverty and food insecurity coincide with the indigenous territories [14].

The national report 2015/2016 by United Nations Development Programme presents the national human development index (NHDI) of Guatemala, which is composed of three fundamental dimensions of the social opportunities: health, education and living standards [11]. From 2000 to 2014, the NHDI had an increment of 17%. This period started with annual rates of increase of 2.3% from 2000 to 2005, which then slowed to 0.3% per year from 2006 to 2014. Improvements in average income and in health stagnated since 2006, which is most likely due to the world financial crisis of 2008, where the economy decreased and has still not fully recovered. In 2014, the health dimension had a lower inequality because the lag is more generalized.

We observed an increase in the proportion of indigenous children in the wealthiest quintile who live in urban areas from 1995 to 2014 (Fig. 1c). The internal migration in Guatemala, mainly from rural areas into towns or cities is slowly but steadily shifting the rural-urban balance of the population [39]. This migration had possibly changed after the signing of the peace accords in 1996, since it led to improvements in the basic infrastructure of services to rural populations, including health and education [40]. Thus, the government leading from 1996 to 2000 linked the peace accords to investments to a reform of the health sector. The most significant improvements in stunting were in the middle wealth tertile, both for indigenous and nonindigenous children. Since in Guatemala approximately 10% of households receive remittances from abroad, and the income due to remittances has become equivalent to about 12% of the national GDP, there is a hypothesis that the increment of remittances in Guatemala could have had a more significant impact in the expansion of the middle class [11] and consequently improvement in the stunting prevalence in under-five children.

Despite the increasing proportion of indigenous population living in urban areas from 1995 to 2014, our findings also confirmed that indigenous are still more rural than urban. The higher levels of prevalence in rural areas is mainly explained by higher levels of poverty, limited access to improved water and sanitation and larger distance to public health services. In addition, indigenous populations who live in especially isolated rural areas or territories face limited access to quality foods in terms of safety, adequacy, and variety [14, 19]. Specifically, health care access is usually worse for indigenous than non-indigenous populations mainly due to language and cultural barriers [41]. But also, indigenous groups present much higher rates of infection than non-indigenous, and these infections are likely to be more severe or more frequently fatal [42, 43].

Maternal nutritional and health status before, during and after pregnancy influence growth and development beginning in the womb [4, 7]. One study showed that Guatemalan women were the shortest of 200 countries in a period of one-hundred years (1914–2014), reaching an average height of 149 cm at 18 years old [44] (2 cm below 50th percentile of WHO standards). A hypothesis proposed by the geneticist James Neel may explain this intergenerational phenomenon, which specifies that an exposure over many generations to poor nutritional conditions may lead to epigenetics mechanisms giving rise to the “thrifty genotype” which would modify the metabolism in order to enhance survival [1]. Nevertheless, intergenerational effects on linear growth could change positively. A community-randomized trial in Guatemala from 1967 to 1977 showed that a nutritious supplement made of a vegetable high-quality protein mixture given to pregnant and lactating women and their children from birth to 7 years old, improved linear growth. Additionally, results from follow-up studies showed an improvement in the height of the next generation, but only in the offspring of girls, and a significant and substantial intergenerational impact on adult human capital and economic productivity [45–47].

A pivotal intervention to struggling child stunting is the improvement of feeding practices (i.e., breastfeeding and complementary feeding) [38], since it is known that children under 2 years old with poor diets (i.e., low in energy, protein and micronutrients) have higher risk of stunting [5]. Guatemalan indigenous children are breastfed for long periods, but complementary feeding begins late and is usually low in protein and micronutrients, being based on staple foods (e.g., tortillas, black beans) and lacking foods from animal sources; consequently, those children also develop micronutrient malnutrition, mainly of iron and zinc [2, 48]. In 2014, 90% of the indigenous population in Guatemala did not have income for a basic basket of goods and services, and 72% could not afford the basic food basket [11]. Victora et al. [49] observed that, even in their first month of life, Guatemalan children were on average − 1.29 SD of height-for-age according to WHO standards, and 24 to 59 months of age children remained below − 2 SD of the reference, which confirms the importance of the first 2 years of life as a window of opportunity to enhance linear growth through adequate feeding practices.

The evidence on the role of conditional cash transfer (CCT) programs on child nutrition is not straightforward [50]. The rationale behind CCT programs is the provision of financial resources to mothers to increase food availability at the household level by reducing out-of-pocket expenditures and opportunity costs [51]. In Guatemala a conditional cash transfer (CCT) program was implemented in 2008, with the purpose to provide bi-monthly cash benefits to the most impoverished families and conditioned them on certain behaviors related to health and education. This social program had different names across subsequent elected governments and suffered modifications over time. Because there was only one evaluation between 2008 and 2012, it is difficult to know the total impact of the CCT program on the reduction of the stunting prevalence [52]. However, we observed that overall stunting prevalence from 2008 to 2014 did not improve substantially. In general, social programs in Guatemala to combat malnutrition do not place sufficient emphasis on the structural causes of food insecurity, but adopt a welfare or “charity” approach. In addition, such programs may fail to reach the most disadvantaged sectors of the population, because the interventions do not overcome the barriers to access services that the poor, indigenous and rural population face [53].

The strengths of this study are that our results were from nationally representative surveys, and as far as we know this is the first comprehensive study of stunting trend prevalence that investigate ethnic inequalities using the five most recent surveys in Guatemala. However, some methodological limitations should be considered. Although a few national surveys used interviewer observation to define ethnicity, which may lead to misclassification, the proportion of the indigenous population estimated from the surveys was similar to what was reported in the last census [54]. Both ethnicity and nutritional assessment are always a challenge in population-based surveys. Ethnicity is subject to limited sample sizes, especially for intersectionality analyses, but we addressed it by analyzing ethnicity according to wealth tertiles instead of quintiles. Regarding nutritional assessment, we observed that the missing values of stunting were not equally distributed across wealth tertiles in all surveys. Despite this drawback, anthropometric results have been used in several publications, including official reports from international organizations, and the percentage of total missing values in each survey was within what has been reported for other nationally representative surveys elsewhere [55, 56].

In conclusion, ethnic group inequities are superimposed upon socioeconomic inequalities in the determination of stunting in Guatemala. Indigenous children are 20 years behind nonindigenous children in relation to stunting prevalence. Poor and rural indigenous children remain as the worst-off group. Therefore, it is essential to 1) evaluate constantly current public nutrition policies in Guatemala (e.g., The National Strategy for the Prevention of Chronic Malnutrition 2016–2020 [57]), whether are being complied as established or need to be adapted; 2) increase the national budget to the different government institutions from the public sector, mainly those related to health and education, and that the budget allocation takes into consideration the indigenous people in order to reduce inequalities in health and nutrition indicators; 3) implement ethnically and culturally appropriate social protection programs that include effective evidence-based interventions [19, 58] with a multisectoral approach to address child malnutrition (i.e., reduce stunting and prevent obesity in children), conduct their corresponding process and impact evaluations with quality, and give continuity to the whole process, regardless of the periods of government; and 4) invest in social inclusion of indigenous population who face the highest risk of undernutrition. Further studies are needed to continue monitoring the prevalence of stunting and ethnic inequalities to reach global targets according to the country context.

## Additional file

Additional file 1: **Table S1.** Proportion of under-five indigenous and nonindigenous children according to place of residence in 1995 and 2014. **Table S2.** Proportion of under-five indigenous and nonindigenous children according to wealth quintiles in 1995 and 2014. **Table S3.** Proportion of under-five indigenous and nonindigenous children according to place of residence and wealth quintiles in 1995 and 2014. **Table S4.** Stunting prevalence in under-five children according to ethnic group, place of residence and wealth quintiles. **Table S5.** Prevalence of stunted indigenous and nonindigenous children under-five according to place of residence and wealth tertiles. (DOCX 38 kb)

## Data Availability

Not applicable

## References

[1] Martorell R (2017). Improved nutrition in the first 1000 days and adult human capital and health. Am J Hum Biol.

[2] Petry N, Olofin I, Boy E, Donahue Angel M, Rohner F (2016). The Effect of Low Dose Iron and Zinc Intake on Child Micronutrient Status and Development during the First 1000 Days of Life: A Systematic Review and Meta-Analysis. Nutrients.

[3] Victora CG, Adair L, Fall C, Hallal PC, Martorell R, Richter L (2008). Maternal and child undernutrition: consequences for adult health and human capital. Lancet..

[4] World Health Organization (2010). Nutrition Landscape Information System (NLIS). Country profile indicators: interpretation guide.

[5] Mal-Ed Network Investigators (2017). Childhood stunting in relation to the pre- and postnatal environment during the first 2 years of life: The MAL-ED longitudinal birth cohort study. PLoS Med.

[6] United Nations Children’s Fund (UNICEF) (2013). Improving Child Nutrition: the achievable imperative for global progress.

[7] de Onis M, Branca F (2016). Childhood stunting: a global perspective. Matern Child Nutr..

[8] World Bank. Country profiles: Guatemala Country profiles. Guatemala; 2018. [updated Apr 16, 2018; cited 2018 March 1, 2018]. Web Page]. Available from: http://data.worldbank.org/country/guatemala

[9] Ministry of Public Health and Social Assistance (MSPAS), National Institute of Statistics (INE), ICF International (2017). National Maternal and Child Health Survey 2014–2015: final report.

[10] Sanchez SM, Scott K, Lopez JH (2016). Guatemala: closing gaps to generate more inclusive growth: world bank.

[11] Programa de las Naciones Unidas para el Desarrollo (2016). Más allá del conflicto, luchas por el bienestar. Informe Nacional de Desarrollo Humano 2015/2016.

[12] Ministerio de Salud Pública y Assistencia Social (MSPAS), Organizatión Panamericana de Salud Publica (OPAS), Organizatión Mundial de la Salud (OPS) (2016). Perfil de salud de los pueblos indígenas de Guatemala.

[13] Ramirez-Zea M, Kroker-Lobos MF, Close-Fernandez R, Kanter R (2014). The double burden of malnutrition in indigenous and nonindigenous Guatemalan populations. Am J Clin Nutr.

[14] Organización de las Naciones Unidas para la Alimentación y la Agricultura, Organización Panamericana de la Salud, Programa Mundial de Alimentos, Fondo de las Naciones Unidas para la Infancia (2018). Panorama de la seguridad alimentaria y nutricional en América Latina y el Caribe.

[15] Tulane University, United Nations Children’s Fund (UNICEF) (2016). Health Equity Report 2016: Analysis of reproductive, maternal, newborn, child and adolescent health inequities in Latin America and the Caribbean to inform policymaking.

[16] World Health Organization (2012). Resolution WHA 65.6. Comprehensive implementation plan on maternal, infant and young child nutrition. Sixty-fifth world health assembly Geneva, 21–26 May 2012.

[17] United Nations (2015). Resolution adopted by the General Assembly on 25 September 2015: Transforming our world: the 2030 Agenda for Sustainable Development.

[18] Commission on Social Determinants of Health (CSDH) (2008). Closing the gap in a generation: health equity through action on the social determinants of health. Final Report of the Commission on Social Determinants of Health.

[19] World Health Organization (2018). Reducing stunting in children: equity considerations for achieving the global nutrition targets 2025.

[20] Paraje G (2009). Child stunting and socio-economic inequality in Latin America and the Caribbean. CEPAL Rev..

[21] Amarante V, Figueroa N, Ullman H (2018). Inequalities in the reduction of child stunting over time in Latin America: evidence from the DHS 2000–2010. Oxf Dev Stud.

[22] de Onis M, Dewey KG, Borghi E, Onyango AW, Blossner M, Daelmans B (2013). The World Health Organization’s global target for reducing childhood stunting by 2025: rationale and proposed actions. Matern Child Nutr.

[23] Ministerio de Salud Publica y Asistencia Social (MPAS), Instituto Nacional de Estadística (INE) (1999). Encuesta Nacional de Salud Materno Infantil 1998–1999.

[24] Ministry of Public Health and Social Assistance, National Institute of Statistics (1996). National Maternal and Child Health Survey 1995.

[25] Ministry of Public Health and Social Assistance (MSPAS), National Institute of Statistics (INE) (2003). National Maternal and Child Health Survey 2002.

[26] Ministry of Public Health and Social Assistance (MSPAS), National Institute of Statistics (INE) (2010). V National Maternal and Child Health Survey 2008–09.

[27] National Institute of Statistics, Ministry of Public Health and Social Assistance (1999). National Maternal and Child Health Survey. 1998–1999.

[28] World Health Organization (2006). WHO Child Growth Standards.

[29] Rutstein SO, The DHS (2008). Wealth index: approaches for rural and urban areas.

[30] Barros AJ, Victora CG (2013). Measuring coverage in MNCH: determining and interpreting inequalities in coverage of maternal, newborn, and child health interventions. PLoS Med.

[31] StataCorp (2013). Stata Statistical Software: Release 13.

[32] Pan American Health Organization (PAHO) (2008). Malnutrition in infants and young children in Latin America and the Caribbean: Achieving the Millennium Development Goals.

[33] Rivera-Dommarco JÁ, Cuevas-Nasu L, González de Cosío T, Shamah-Levy T, García-Feregrino R (2013). Desnutrición crónica en México en el último cuarto de siglo: análisis de cuatro encuestas nacionales. salud pública de méxico.

[34] Restrepo-Méndez MC, Barros AJ, Black RE, Victora CG (2015). Time trends in socio-economic inequalities in stunting prevalence: analyses of repeated national surveys. Public Health Nutr.

[35] Huicho L, Huayanay-Espinoza CA, Herrera-Perez E, Segura ER, de Guzman JN, Rivera-Ch M (2017). Factors behind the success story of under-five stunting in Peru: a district ecological multilevel analysis. BMC Pediatr.

[36] Peru, Ministerio de Economía y Finanzas, Instituto Nacional de Estadística e Informática (2019). Perú: Indicadores de resultados de los programas presupuestales, 2013-2018. Encuesta demográfica y de salud familiar.

[37] Huicho L, Segura ER, Huayanay-Espinoza CA, de Guzman JN, Restrepo-Méndez MC, Tam Y (2016). Child health and nutrition in Peru within an antipoverty political agenda: a countdown to 2015 country case study. Lancet Glob Health.

[38] Martorell R (2012). Intervenciones y opciones de políticas Para combatir la desnutrición en Guatemala title: intervention and policy options for combating malnutrition in Guatemala.

[39] Lindstrom DP, Hernández CH (2006). Internal migration and contraceptive knowledge and use in Guatemala. Int Fam Plan Perspect.

[40] Republica de Guatemala, Presidencia de la República, Secretaría de la Paz (1996). Los Acuerdos de Paz en Guatemala.

[41] Valeggia CR, Snodgrass JJ (2015). Health of indigenous peoples. Annu Rev Anthropol.

[42] Gracey M, King M (2009). Indigenous health part 1: determinants and disease patterns. Lancet.

[43] Mesenburg MA, Restrepo-Mendez MC, Amigo H, Balandrán AD, Barbosa-Verdun MA, Caicedo-Velásquez B (2018). Ethnic group inequalities in coverage with reproductive, maternal and child health interventions: cross-sectional analyses of national surveys in 16 Latin American and Caribbean countries. Lancet Glob Health.

[44] Collaboration NRF (2016). A century of trends in adult human height. Elife..

[45] Martorell R, Melgar P, Maluccio JA, Stein AD, Rivera JA (2010). The nutrition intervention improved adult human capital and economic productivity. J Nutr.

[46] Martorell R, Zongrone A (2012). Intergenerational influences on child growth and undernutrition. Paediatr Perinat Epidemiol.

[47] Ramirez-Zea M, Melgar P, Rivera JA (2010). INCAP Oriente longitudinal study: 40 years of history and legacy. J Nutr.

[48] Solomons N, Vossenaar M (2013). Nutrient density in complementary feeding of infants and toddlers. Eur J Clin Nutr.

[49] Victora CG, de Onis M, Hallal PC, Blossner M, Shrimpton R (2010). Worldwide timing of growth faltering: revisiting implications for interventions. Pediatrics..

[50] Black RE, Victora CG, Walker SP, Bhutta ZA, Christian P, de Onis M (2013). Maternal and child undernutrition and overweight in low-income and middle-income countries. Lancet..

[51] Hoddinott J, Bassett L (2008). Conditional cash transfer programs and nutrition in Latin America: assessment of impacts and strategies for improvement.

[52] Sandberg J, Tally E (2015). Politicisation of conditional cash transfers: the case of Guatemala. Dev Policy Rev.

[53] Center for Economic and Social Rights/Instituto Centroamericano de Estudios Fiscales (2009). ¿Derechos o privilegios? El compromiso fiscal con la salud, la educación y la alimentación en Guatemala.

[54] Republica de Guatemala, Instituto Nacional de Estadística, Censos Nacionales XI de Poblacion y VI de Habitación 2002 (2003). Características de la Población y de los Locales de Habitación Censados.

[55] Pullum TW (2008). An assesment of the quality of data on health and nutrition in the DHS surveys, 1993–2003. DHS methodological reports no. 6.

[56] Shireen A, Kothari MT, Pullum T (2015). An assesment of the quality of DHS antropometric data, 2005–2014. DHS methodological reports no. 16.

[57] Comisión Nacional para la Reducción de la Desnutrición Crónica (2016). Estrategia Nacional para la prevención de la desnutrición crónica 2016-2020.

[58] United Nations International Children’s Emergency Fund (2017). Reducing stunting in children under five years of age: a comprehensive evaluation of UNICEF's strategies and program performance. Global synthesis report.

