# Supplementary material for: Ethnic inequalities and trends in stunting prevalence among Guatemalan children: an analysis using national health surveys 1995–2014
Source: Int J Equity Health. 2019 Jul 18;18:110. doi: 10.1186/s12939-019-1016-0 (PMC6639956; doi:10.1186/s12939-019-1016-0)
Supplement: Supplementary file 1 — Table S1. Proportion of under-five indigenous and nonindigenous children according to place of residence in 1995 and 2014. Table S2. Proportion of under-five indigenous and nonindigenous children according to wealth quintiles in 1995 and 2014. Table S3. Proportion of under-five indigenous and nonindigenous children according to place of residence and wealth quintiles in 1995 and 2014. Table S4. Stunting prevalence in under-five children according to ethnic group, place of residence and wealth quintiles. Table S5. Prevalence of stunted indigenous and nonindigenous children under-five according to place of residence and wealth tertiles. (DOCX 38 kb) [file 12939_2019_1016_MOESM1_ESM.docx]

**Additional file 1**

**Table S1** Proportion of under-five indigenous and nonindigenous children according to place of residence in 1995 and 2014

| **Ethnic group** | **Year** | **Urban** | | | **Rural** | | |
| --- | --- | --- | --- | --- | --- | --- | --- |
|  |  | **Weighted**  **N** | **%** | **95% CI** | **Weighted N** | **%** | **95% CI** |
| Indigenous | 1995 | 745 | 19.1 | (15.7; 23.0) | 3164 | 80.9 | (77.0; 84.3) |
|  | 2014 | 1459 | 25.6 | (22.2; 29.2) | 4247 | 74.4 | (70.8; 77.8) |
|  |  |  |  |  |  |  |  |
| Nonindigenous | 1995 | 2138 | 40.8 | (37.2; 44.5) | 3104 | 59.2 | (55.5; 62.8) |
|  | 2014 | 2881 | 44.2 | (41.4; 47.1) | 3633 | 55.8 | (52.9; 58.6) |

**Table S2** Proportion of under-five indigenous and nonindigenous children according to wealth quintiles in 1995 and 2014

| **Ethnic group** | **Year** | **Poorest** | | | **2^nd^** | | | **3^rd^** | | | **4^th^** | | | **Wealthiest** | | |
| --- | --- | --- | --- | --- | --- | --- | --- | --- | --- | --- | --- | --- | --- | --- | --- | --- |
|  |  | **Weighted**  **N** | **%** | **95% CI** | **Weighted**  **N** | **%** | **95% CI** | **Weighted**  **N** | **%** | **95% CI** | **Weighted**  **N** | **%** | **95% CI** | **Weighted**  **N** | **%** | **95% CI** |
| Indigenous | 1995 | 1558 | 39.8 | (35.2; 44.6) | 1120 | 28.6 | (25.4; 32.1) | 775 | 19.8 | (17.2; 22.8) | 383 | 9.8 | (7.9; 12.0) | 74 | 1.9 | (1.3; 2.8) |
|  | 2014 | 2311 | 40.5 | (37.0; 44.1) | 1529 | 26.8 | (24.5; 29.3) | 1004 | 17.6 | (15.7; 19.7) | 581 | 10.2 | (8.7; 11.9) | 282 | 4.9 | (3.9; 6.2) |
|  |  |  |  |  |  |  |  |  |  |  |  |  |  |  |  |  |
| Nonindigenous | 1995 | 916 | 17.5 | (14.8; 20.5) | 1009 | 19.3 | (16.4; 22.4) | 1115 | 21.3 | (18.7; 24.1) | 1213 | 23.1 | (20.2; 26.3) | 989 | 18.9 | (15.8; 22.3) |
|  | 2014 | 948 | 14.6 | (12.8; 16.5) | 1193 | 18.3 | (16.8; 19.9) | 1415 | 21.7 | (20.1; 23.4) | 1595 | 24.5 | (22.7; 26.4) | 1363 | 20.9 | (19.1; 22.9) |

**Supplementary Table 3.** Proportion of under-five indigenous and nonindigenous children according to place of residence and wealth quintiles in 1995 and 2014

| **Ethnic group** | **Place of residence** | **Year** | **Poorest** | | | **2^nd^** | | | **3^rd^** | | | **4^th^** | | | **Wealthiest** | | |
| --- | --- | --- | --- | --- | --- | --- | --- | --- | --- | --- | --- | --- | --- | --- | --- | --- | --- |
|  |  |  | **Weighted**  **N** | **%** | **95% CI** | **Weighted**  **N** | **%** | **95% CI** | **Weighted**  **N** | **%** | **95% CI** | **Weighted**  **N** | **%** | **95% CI** | **Weighted**  **N** | **%** | **95% CI** |
| Indigenous | Rural | 1995 | 1464 | 46.3 | (41.1; 51.5) | 974 | 30.8 | (26.8; 35.0) | 513 | 16.2 | (13.9; 18.9) | 197 | 6.2 | (4.4; 8.7) | 17* | 0.5 | (0.2; 1.3) |
|  |  | 2014 | 2062 | 48.5 | (44.4; 52.7) | 1245 | 29.3 | (26.5; 32.4) | 664 | 15.6 | (13.4; 18.1) | 220 | 5.2 | (3.8; 7.0) | 56 | 1.3 | (0.6; 2.8) |
|  |  |  |  |  |  |  |  |  |  |  |  |  |  |  |  |  |  |
|  | Urban | 1995 | 93 | 12.5 | (7.6; 19.9) | 146 | 19.6 | (15.8; 24.1) | 263 | 35.2 | (27.9; 43.4) | 186 | 25.0 | (19.6; 31.2) | 57 | 7.6 | (4.8; 11.9) |
|  |  | 2014 | 249 | 17.1 | (11.5; 24.6) | 284 | 19.5 | (15.9; 23.6) | 339 | 23.3 | (20.0; 26.9) | 361 | 24.7 | (20.9; 29.0) | 226 | 15.5 | (11.9; 19.8) |
|  |  |  |  |  |  |  |  |  |  |  |  |  |  |  |  |  |  |
| Nonindigenous | Rural | 1995 | 828 | 26.7 | (22.9; 30.8) | 875 | 28.2 | (24.8; 31.9) | 736 | 23.7 | (21.0; 26.6) | 485 | 15.6 | (12.5; 19.4) | 182 | 5.8 | (3.5; 9.5) |
|  |  | 2014 | 849 | 23.4 | (20.6; 26.4) | 986 | 27.1 | (24.9; 29.5) | 949 | 26.1 | (23.9; 28.5) | 636 | 17.5 | (15.4; 19.7) | 215 | 5.9 | (4.7; 7.3) |
|  |  |  |  |  |  |  |  |  |  |  |  |  |  |  |  |  |  |
|  | Urban | 1995 | 88 | 4.1 | (1.8; 9.0) | 135 | 6.3 | (3.0; 12.7) | 379 | 17.7 | (13.1; 23.6) | 728 | 34.1 | (29.3; 39.1) | 808 | 37.8 | (31.9; 44.1) |
|  |  | 2014 | 99 | 3.4 | (1.9; 6.1) | 207 | 7.2 | (5.7; 9.1) | 466 | 16.2 | (14.0; 18.7) | 959 | 33.3 | (30.6; 36.1) | 1149 | 39.9 | (36.0; 43.8) |

*Sample size < 25 observations

**Table S4** Stunting prevalence in under-five children according to ethnic group, place of residence and wealth quintiles

| **Stratifiers** | **DHS 1995** | | **DHS 1998** | | **RHS 2002** | | **RHS 2008** | | **DHS 2014** | |
| --- | --- | --- | --- | --- | --- | --- | --- | --- | --- | --- |
|  | **Weighted N** | **% (95% CI)** | **Weighted N** | **% (95% CI)** | **Weighted N** | **% (95% CI)** | **Weighted N** | **% (95% CI)** | **Weighted N** | **% (95% CI)** |
| Ethnic group |  |  |  |  |  |  |  |  |  |  |
| Indigenous | 3296 | 72.7 (70.5; 74.7) | 1333 | 73.6 (70.0; 76.9) | 1877 | 75.0 (72.8; 77.0) | 3876 | 63.6 (61.8; 65.5) | 5603 | 61.1 (58.8; 63.5) |
| Non-indigenous | 4487 | 43.0 (39.9; 46.1) | 2205 | 42.1 (37.5; 47.0) | 2746 | 41.2 (38.7; 43.8) | 4607 | 34.9 (33.2; 36.7) | 6405 | 33.9 (31.9; 35.9) |
|  |  |  |  |  |  |  |  |  |  |  |
| Place of residence |  |  |  |  |  |  |  |  |  |  |
| Rural | 5355 | 62.6 (60.6; 64.6) | 2317 | 61.8 (56.9; 66.3) | 3109 | 61.1 (58.9; 63.2) | 5362 | 56.7 (55.1; 58.3) | 8135 | 53.0 (50.8; 55.1) |
| Urban | 2454 | 40.2 (36.0; 44.5) | 1221 | 39.2 (30.6; 48.6) | 1514 | 42.1 (38.7; 45.6) | 3120 | 33.2 (31.1; 35.3) | 4431 | 34.6 (31.9; 37.4) |
|  |  |  |  |  |  |  |  |  |  |  |
| Wealth quintiles |  |  |  |  |  |  |  |  |  |  |
| Poorest | 2097 | 70.5 (67.7; 73.1) | 946 | 71.3 (66.0; 76.2) | 1303 | 74.1 (71.7; 76.3) | 2591 | 67.9 (65.8; 69.9) | 3357 | 65.9 (63.3; 68.4) |
| 2^nd^ | 1785 | 67.2 (64.4; 70.0) | 757 | 69.0 (64.5; 73.2) | 1090 | 64.9 (61.3; 68.3) | 1995 | 57.7 (55.1; 60.3) | 2809 | 57.1 (54.4; 59.8) |
| 3^rd^ | 1635 | 58.9 (55.3; 62.5) | 704 | 58.2 (49.2; 66.6) | 1007 | 47.4 (43.6; 51.2) | 1660 | 41.7 (38.7; 44.8) | 2476 | 43.8 (41.0; 46.6) |
| 4^th^ | 1425 | 39.3 (34.2; 44.7) | 701 | 34.0 (28.6; 40.0) | 762 | 40.0 (35.0; 45.2) | 1452 | 24.8 (21.8; 28.2) | 2260 | 28.9 (26.4; 31.4) |
| Wealthiest | 866 | 15.8 (12.2; 20.4) | 430 | 15.0 (11.1; 20.1) | 461 | 18.1 (12.8; 24.8) | 784 | 14.3 (11.2; 18.0) | 1665 | 17.4 (15.1; 19.9) |
|  |  |  |  |  |  |  |  |  |  |  |

**Table S5** Prevalence of stunted indigenous and nonindigenous children under-five according to place of residence and wealth tertiles

| **Ethnic group** |  |  | **Place of residence** | | | |  | **Wealth Tertiles** | | | | | |
| --- | --- | --- | --- | --- | --- | --- | --- | --- | --- | --- | --- | --- | --- |
|  | **Year** |  | **N** | **Rural**  **% (95% CI)** | **N** | **Urban**  **% (95% CI)** |  | **N** | **Poorest**  **% (95% CI)** | **N** | **2^nd^**  **% (95% CI)** | **N** | **Wealthiest**  **% (95% CI)** |
| Indigenous | 1995 |  | 2681 | 73.6 (71.4; 75.7) | 615 | 68.7 (62.5; 74.3) |  | 2060 | 74.3 (71.7; 76.7) | 1082 | 73.4 (70.0; 76.4) | 154 | 46.4 (35.5; 57.6) |
|  | 1998 |  | 1060 | 74.1 (69.9; 77.8) | 272 | 71.8 (62.7; 79.3) |  | 899 | 73.7 (69.3; 77.7) | 386 | 76.5 (68.4; 83.0) | 48** | 47.9 (25.4; 71.3) |
|  | 2002 |  | 1392 | 78.3 (76.2; 80.2) | 485 | 65.5 (60.0; 70.6) |  | 1234 | 80.4 (78.4; 82.3) | 527 | 68.6 (63.5; 73.2) | 115 | 45.9 (36.6; 55.5) |
|  | 2008 |  | 2861 | 67.7 (65.5; 69.8) | 1015 | 52.3 (48.8; 55.8) |  | 2348 | 70.7 (68.5; 72.8) | 1183 | 58.3 (54.8; 61.8) | 345 | 33.9 (28.2; 40.2) |
|  | 2014 |  | 4172 | 63.8 (61.0; 66.5) | 1430 | 53.4 (48.8; 58.0) |  | 3768 | 67.1 (64.3; 69.7) | 1561 | 51.5 (47.7; 55.3) | 274 | 34.8 (27.7; 42.7) |
|  |  |  |  |  |  |  |  |  |  |  |  |  |  |
| Nonindigenous | 1995 |  | 2653 | 51.6 (48.1; 55.0) | 1834 | 30.6 (25.6; 36.0) |  | 1479 | 62.9 (59.3; 66.3) | 1664 | 44.7 (41.4; 48.1) | 1344 | 18.9 (15.6; 22.8) |
|  | 1998 |  | 1257 | 51.4 (45.2; 57.5) | 948 | 29.9 (22.4; 38.6) |  | 709 | 66.2 (60.6; 71.4) | 790 | 42.4 (36.9; 48.1) | 706 | 17.7 (13.4; 22.8) |
|  | 2002 |  | 1716 | 47.2 (44.0; 50.4) | 1029 | 31.2 (27.3; 35.3) |  | 992 | 59.9 (56.2; 63.4) | 1081 | 37.2 (33.3; 41.3) | 673 | 20.1 (15.7; 25.2) |
|  | 2008 |  | 2502 | 44.2 (41.8; 46.6) | 2105 | 23.9 (21.4; 26.6) |  | 1222 | 56.5 (53.4; 59.5) | 1649 | 37.1 (34.1; 40.2) | 1736 | 17.7 (15.1; 20.6) |
|  | 2014 |  | 3593 | 40.5 (37.7; 43.3) | 2812 | 25.4 (22.9; 28.0) |  | 2121 | 52.9 (49.6; 56.2) | 2966 | 28.9 (26.7; 31.3) | 1318 | 14.3 (12.1; 16.8) |

**Sample size between 25 and 50 observations
